# Optimizing oropharyngeal swabbing techniques: the relationship between force applied and SARS-CoV-2 detection sensitivity

**DOI:** 10.3205/dgkh000539

**Published:** 2025-03-07

**Authors:** Peter Melcher, Corinna Pietsch, Sandra Bergs, Yasmin Youssef, Paul Rahden, Pierre Hepp, Ralf Henkelmann

**Affiliations:** 1 Department of Orthopedics, Trauma and Plastic Surgery, University of Leipzig, Leipzig, Germany; 2 Department of Orthopedics, Trauma Surgery, Helios Hospital Leisnig, Leisnig, Germany; 3 Institute of Medical Microbiology and Virology, University of Leipzig, Leipzig, Germany; 4 Department of Implementation Research, Bernhard Nocht Institute for Tropical Medicine, Hamburg, Germany

**Keywords:** SARS-CoV-2, NAT, preanalytical factors, oropharyngeal swab, sample force, cell count

## Abstract

**Aim:**

Nasopharyngeal and oropharyngeal swabs are essential for diagnosing SARS-CoV-2 infections, with nucleic acid testing (NAT) being the most sensitive method. However, NAT results are heavily influenced by preanalytical factors, including quality of the sample. This study examines the effect of applied force during oropharyngeal sampling on sample quality, specifically assessing cell count and the associated NAT cycle threshold (Ct) values.

**Methods:**

A three-phase investigation was conducted to explore the relationship between sampling force and cell quantity, as well as the impact of cell count on NAT sensitivity.

**Results:**

A significantly lower Ct value was achieved by artificially increasing the cell count in a swab sample and applying a greater force resulted in higher cell counts, but the opposite effect on Ct values of SARS-CoV-2 NAT was shown.

**Conclusion:**

These findings indicate that while applying greater force during sample collection increases the number of collected cells, it does not improve the sensitivity of SARS-CoV-2 detection and can even lead to poorer results. Further research should focus on optimizing swab design to improve sample quality and the number of cells obtained.

## Zusammenfassung

**Ziel:**

Nasopharyngeale und oropharyngeale Abstriche sind entscheidend für die Diagnose von SARS-CoV-2-Infektionen, wobei die Nukleinsäuretestung (NAT) die empfindlichste Methode darstellt. Die NAT-Ergebnisse werden jedoch stark durch präanalytische Faktoren beeinflusst, einschließlich der Qualität der Probe. Diese Studie untersucht den Einfluss der angewendeten Kraft während deroropharyngealen Probenentnahme auf die Probenqualität, insbesondere durch die Bewertung der Zellzahl und der damit verbundenen NAT-Zyklus-Schwellenwerte (Ct-Werte).

**Methode:**

Eine dreiphasige Untersuchung wurde durchgeführt, um die Beziehung zwischen Entnahmekraft und Zellmenge sowie den Einfluss der Zellzahl auf die NAT-Empfindlichkeit zu untersuchen.

**Ergebnisse:**

Während ein signifikant niedrigerer Ct-Wert durch künstliche Erhöhung der Zellzahl in einer Abstrichprobe erreicht werden konnte und die Anwendung größerer Kraft zu einer höheren Zellzahl führte, konnte ein gegenteiliger Effekt auf die Ct-Werte der SARS-CoV-2-NAT nachgewiesen werden.

**Schlussfolgerung:**

Die Ergebnisse zeigen, dass, obwohl die Anwendung größerer Kraft während der Probenentnahme die Anzahl der gesammelten Zellen erhöht, das die Empfindlichkeit der SARS-CoV-2-Erkennung nicht verbessert und sogar zu schlechteren Ergebnissen führen kann. Weitere Untersuchungen sollten sich darauf konzentrieren, das Design der Abstriche zu optimieren, um die Probenqualität und die Anzahl der gewonnenen Zellen zu verbessern.

## Introduction

Oropharyngeal and nasopharyngeal swabs are critical diagnostic tools that played an essential role in testing strategies during the COVID-19 pandemic for identifying individuals infected with SARS-CoV-2. Numerous tests have been developed to detect SARS-CoV-2, with nucleic acid testing (NAT) being the most sensitive and specific for confirming infection [1]. However, the accuracy of NAT is highly dependent on the quality of the collected sample, which has led the WHO to publish guidelines on obtaining optimal test procedure for SARS-CoV-2 testing [2].

NAT results can vary not only intraindividually but also throughout the different stages of infection. Since the cycle threshold (Ct) is often used to assess viral load and estimate the risk of transmission, as well as the need for quarantine, obtaining reliable results is crucial. Despite this, few studies have focused on improving preanalytical conditions to enhance sample quality [3].

This study investigates whether increasing the applied force during oropharyngeal sampling improves preanalytical quality for detecting SARS-CoV-2. The methodology is based on findings from our previous feasibility study [4].

## Methods

The study was conducted in three phases to investigate the correlation between cell count, sampling force and Ct values in the detection of SARS-CoV-2. 

In the first phase, 60 oropharyngeal swabs were manually collected from hospitalized patients, excluding those in the ICU, with confirmed SARS-CoV-2 infections at various stages of the disease, following the WHO and Robert Koch Institute (RKI) guidelines [5]. The hypothesis was that higher cell counts could lead to more accurate NAT results.

To assess cell count and sample quality, all swabs were vortexed for 15 seconds to ensure thorough suspension of cells in the swab medium. Subsequently, 800 µl of the swab medium was centrifuged at 300g for 5 minutes, separating the sample into a cell-poor supernatant and a cell-rich pellet. From the supernatant, 500 µl was carefully removed, and 200 µl was used for nucleic acid extraction with the Roche MagNA Pure 96 DNA and Viral NA Small Volume Kit (Roche, Heidelberg, Germany). The cell-rich pellet was resuspended in the remaining 300 µl of supernatant, and 200 µl of this resuspension was also used for RNA extraction. The extracted nucleic acids were eluted in 100 µl of elution buffer.

To quantify viral RNA, 35 µl of the prepared nucleic acid elution was tested using the Abbott RealTime SARS-CoV-2 Assay (Abbott, Wiesbaden, Germany). For cell count assessment, copies of the human RNase P gene in 5 µl of nucleic acid elution were quantified on a LightCycler 2.0 instrument (Roche, Heidelberg, Germany), following WHO recommendations [6]. Based on the detected RNase P copies, the total cell count was calculated, assuming a diploid chromosome set. Eleven patients who achieved a negative test result for cell-poor and cell-rich fraction were no longer considered positive and were not included in the data analysis.

In phase two, a force-feedback device was utilized to obtain oropharyngeal samples from healthy individuals free of SARS-CoV-2 infection. This was done to examine the correlation between the applied force and the quantity of cells collected in the samples. A total of n=15 samples were collected at each force setting of 1.5 N, 2.5 N, and 3.5 N. These force levels had previously been evaluated in a study as well-tolerated during oropharyngeal swabbing [4]. On the day of acquisition, each swab was vortexed for 15 seconds to ensure thorough suspension of cells in the swab medium, facilitating the assessment of cell count and sample quality. Subsequently, 200 µl of the swab medium was used for nucleic acid extraction and cell count analysis as described above.

In phase three, samples were obtained from hospitalized patients with confirmed SARS-CoV-2 infection at various stages of the disease, excluding patients in the ICU. Samples were taken using controlled forces of 1.5 N, 2.5 N, and 3.5 N as described above. On the day of acquisition, all three swabs were vortexed for 15 seconds to ensure thorough suspension of cells in the swab medium. Subsequently, 200 µl of swab medium was used for nucleic acid extraction and assessment of SARS-CoV-2 RNA, as described previously. Seven patients who achieved a negative test result for all three forces were no longer considered positive and were not included in the data analysis.

Data documentation was conducted using Microsoft Excel, while statistical analysis was performed with SPSS 29 (IBM). Findings are reported as the mean value and standard deviation for continuous data and number with percentage for categorical data. For the statistical analysis, the Wilcoxon test was used, with statistical significance defined as p<0.05. Two-sided testing was conducted in phase one. In the presence of a detectable trend in one direction, the statistical analysis in phases two and three was conducted using a one-sided Wilcoxon test. Negative test results in NAT were assigned a Ct value of 45, which corresponds to the detection limit of the NAT used. The figures were generated using both SPSS and Microsoft Excel.

Informed consent was obtained from all patients prior to conduction of any study procedure including nasal swabs. The study was approved by Ethics Committee of the Faculty of Medicine at Leipzig University (reference number: 057/20-ek).

## Results

**Figure 1:**
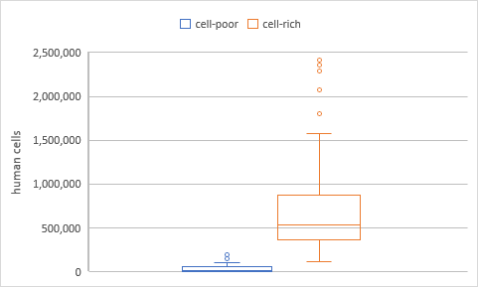
Calculated cell counts in the cell-poor and cell-rich fractions of samples after centrifugation

The investigations in phase one aimed to examine the difference between Ct values in samples of high and low cell counts. Therefore, all samples were centrifuged. This led to a significantly higher cell count in the two fractions of the samples. The cell-poor supernatant showed a mean calculated cell count of 44,444±62,518 cells, while the cell-rich fraction showed a significantly higher mean calculated cell-count of 693,908±453,012 (p<0.01; Figure 1 (Fig. 1)). 

**Figure 2:**
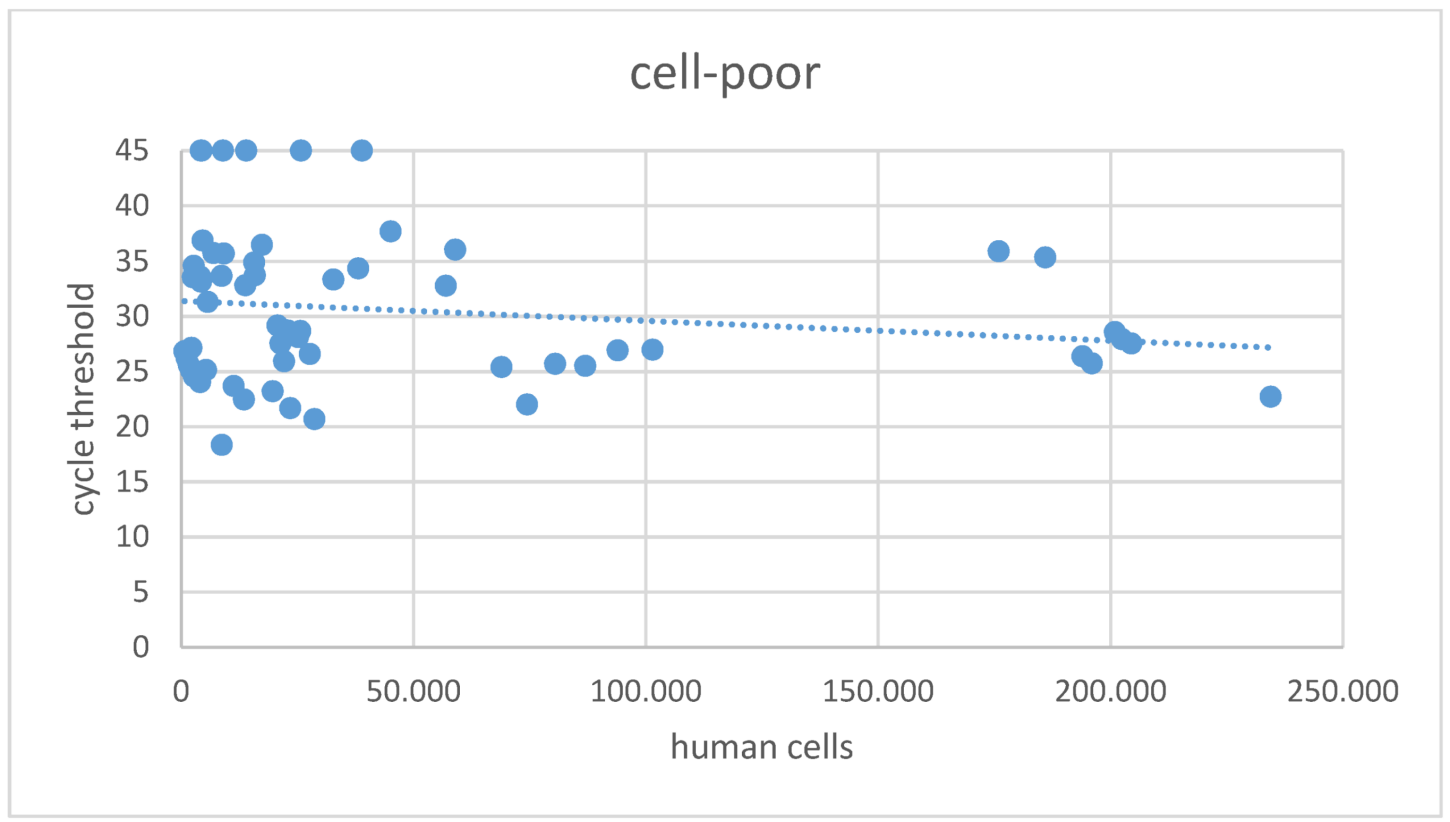
Cycle threshold values in SARS-CoV-2 NAT for the cell-poor fraction of samples following centrifugation

**Figure 3:**
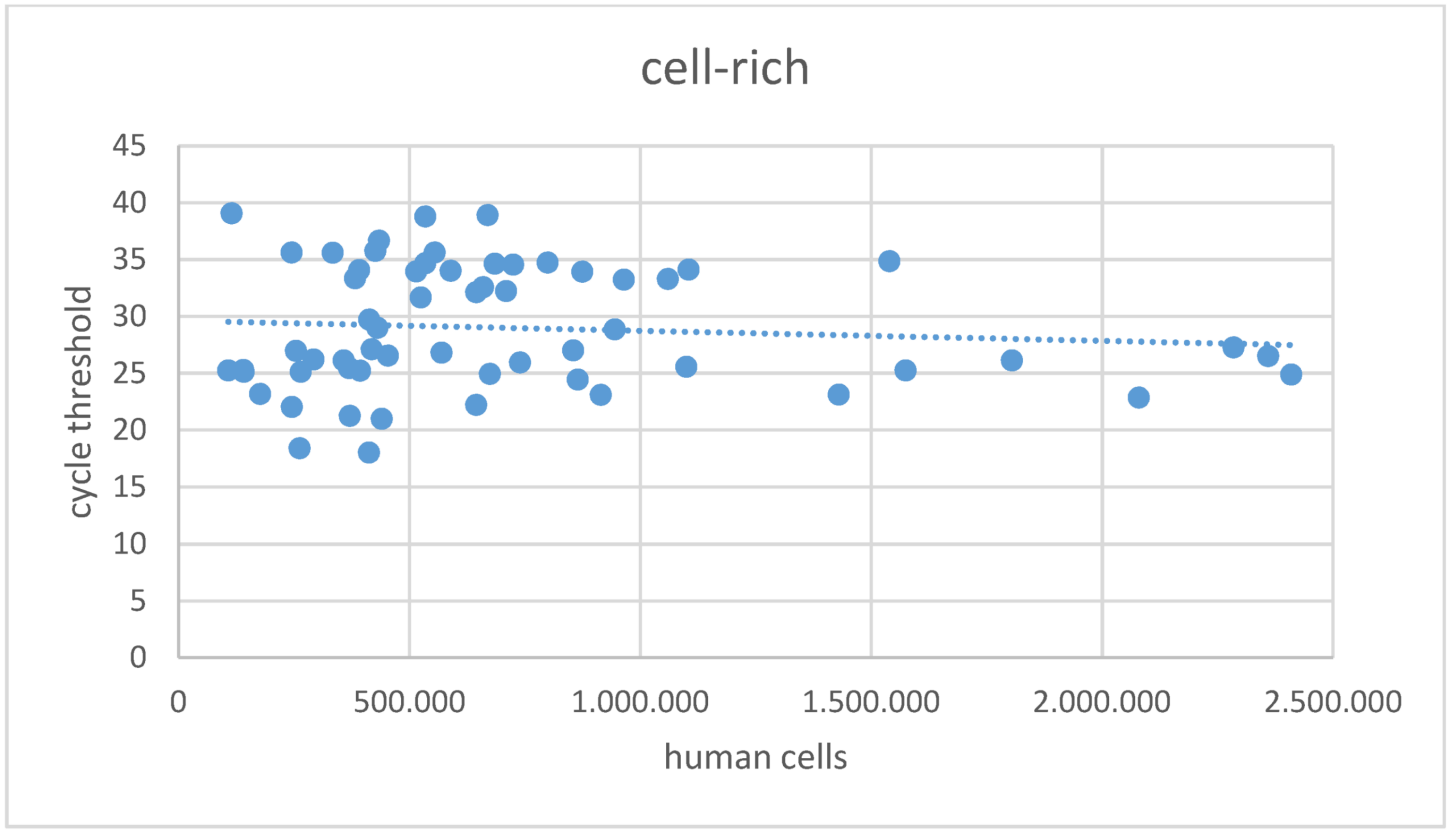
Cycle threshold values in SARS-CoV-2 NAT for the cell-rich fraction of samples following centrifugation

The overall mean Ct value in the cell-poor fraction was 30.8±7.0 (Figure 2 (Fig. 2)), with 18 samples in this fraction showing a Ct >45 in SARS-CoV-2 NAT. In the cell-rich fraction, the mean Ct value was 29.0±5.4 (Figure 3 (Fig. 3)), and 11 samples exhibited a Ct >45. Samples reporting Ct values >45 in both the cell-poor and cell-rich fractions were considered SARS-CoV-2 RNA negative. A Wilcoxon test was conducted, yielding a p-value <0.001. 

**Figure 4:**
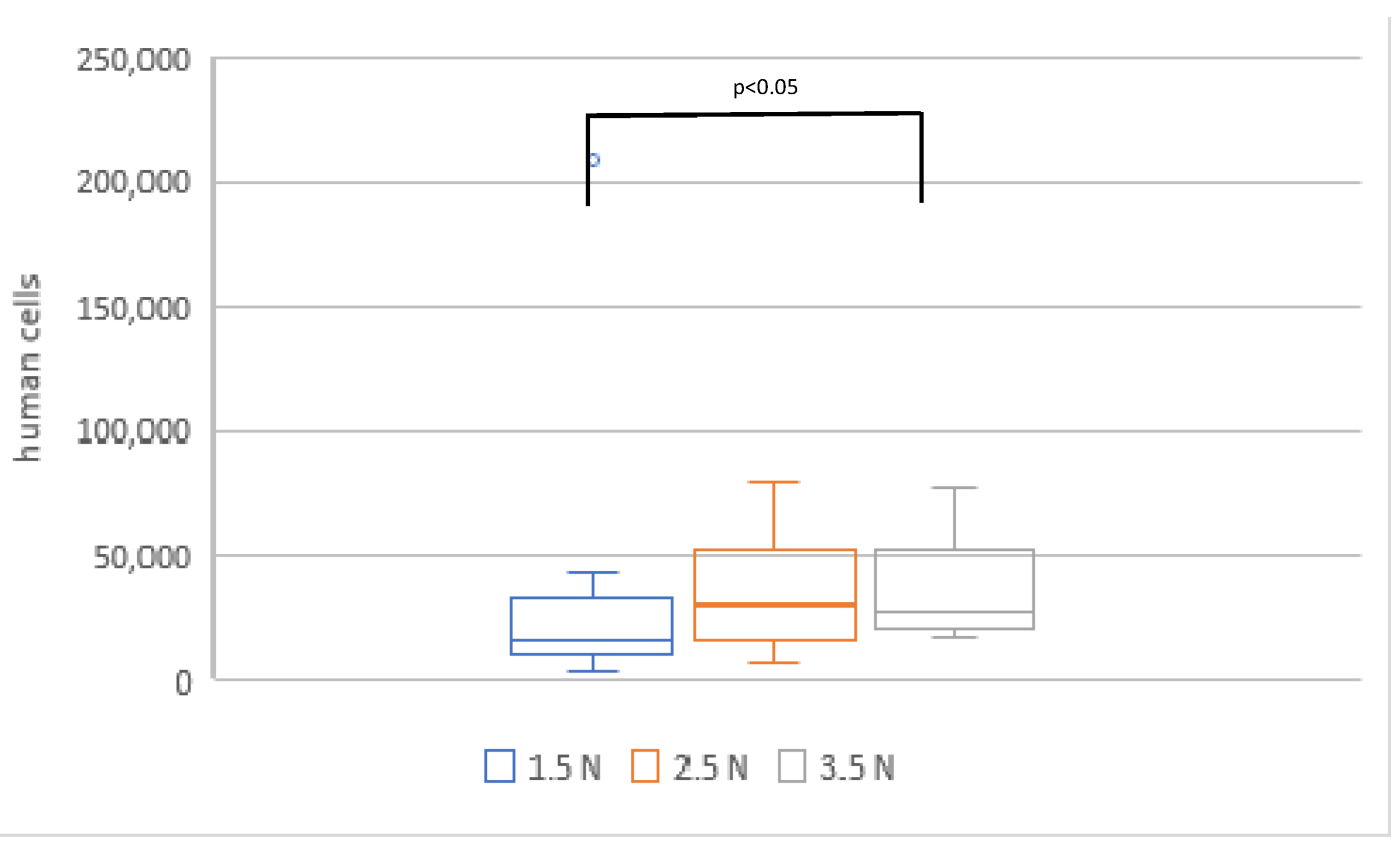
Comparison of calculated cell counts in samples collected with forces of 1.5 N, 2.5 N, and 3.5 N

Phase two investigated the correlation between sampling force and the number of cells collected. A total of 15 samples were collected for each force level, although one sample of the 2.5 N group was lost during transportation to the lab, resulting in n=15 for the 1.5 N group, n=14 for the 2.5 N group and n=15 for the 3.5 N group. The average number of cells collected was calculated at 31,141±50,685 for the 1.5 N group, 35,467±20,723 for the 2.5 N group, and 36,313±18,389 for the 3.5 N group (Figure 4 (Fig. 4)). A significantly higher calculated cell count was observed in samples taken with a force of 3.5 N compared to those taken with 1.5 N (p<0.05). No statistically significant differences were found between the other groups. 

**Figure 5:**
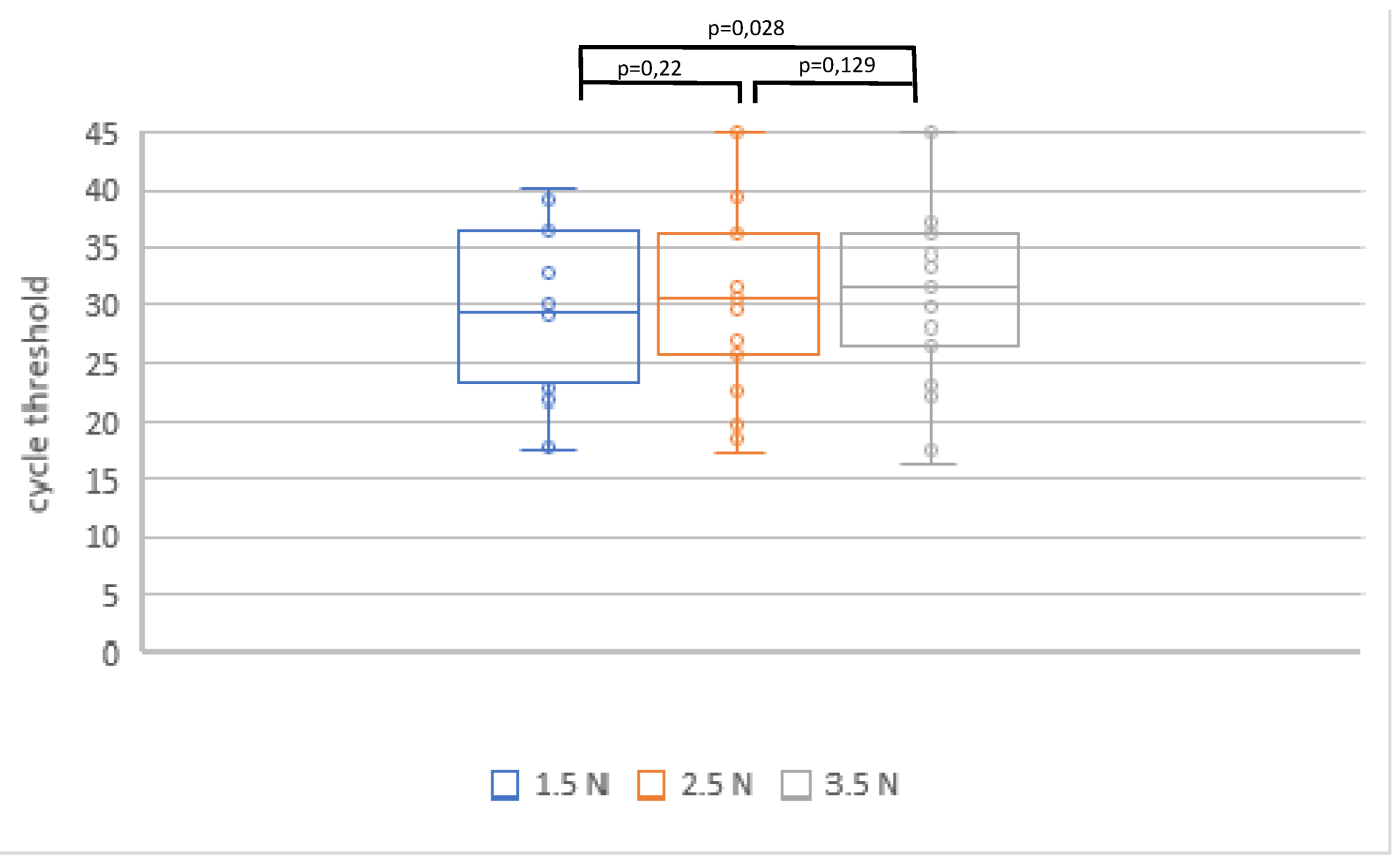
Ct values in SARS-CoV-2 NAT in relation to varying swabbing forces in SARS-CoV-2 positive patients

In phase three, samples were collected from 30 patients and 23 were analyzed in the 1.5 N, 2.5 N and 3.5 N groups. Samples of seven patients were considered negative, with Ct values >45 in all three analyses of the SARS-CoV-2 NAT. The mean Ct values were 29.5±7.1 in the 1.5 N group, 30.4±8.2 in the 2.5 N group, and 31.4±8.5 in the 3.5 N group (Figure 5 (Fig. 5)). A statistically significant difference was found between the swab force of 1.5 N and 3.5 N showing less diagnostic precision in the 3.5 N group.

## Discussion

Multiple SARS-CoV-2 tests are available, including oropharyngeal and nasopharyngeal NAT. Additionally, some NATs use swab samples from the mouth, nasal atrium, or a combination of both. There are also various antigen rapid diagnostic tests, each with differing levels of specificity of the sensitivity. These antigen tests are most sensitive during the first week after symptom onset and in patients with high viral load (Ct <25) [7].

Nasopharyngeal swabs are considered the gold standard for SARS-CoV-2 testing, but oropharyngeal swabs offer comparable sensitivity and are more comfortable for patients [8].

Since SARS-CoV-2 replication depends on host cells, a higher cell count in the swab is expected to improve sensitivity. Although SARS-CoV-2 can be detected in cell-poor swabs, a higher cell count is associated with significantly lower Ct values. This raises the question of how to increase the number of cells collected during swabbing. Previous research demonstrated that the swabbing technique affects both cell count and Ct value [9]. As shown in phase 2 of our study, applying greater force during oropharyngeal sampling also significantly increases the number of cells collected per sample. 

However, we were unable to confirm the effect with swabs of SARS-CoV-2-positive patients. Increasing the force during an oropharyngeal SARS-CoV-2 swab did not improve the sensitivity of the test. In contrast to the null hypothesis, the comparison of the means showed an increase in the Ct values with increasing force and even a significant difference when comparing the 1.5 N group with the 3.5 N group. One possible explanation is that even at the highest applied force (36,313±18,389 cells), the cell count did not approach the levels achieved through centrifugation (693,908±453,012 cells). Additionally, an RNase P assay does not provide information about the types of cells collected during swabbing, and increased swabbing force may lead to a higher proportion of non-SARS-CoV-2-infected cells. Applying more force can also be uncomfortable for patients and may result in rushed or less precise swabbing, limiting the potential benefits of increased force. Further research is needed to improve swab quality, as modification in swab design could enhance sensitivity without compromising patient comfort. 

In summary, using stronger forces during oropharyngeal swab sampling for SARS-CoV-2 does not enhance test sensitivity. Contrary to the basic assumption, it must be concluded that swabs taken with 3.5 N result in a higher Ct value and can thus influence the swab result. A force of 1.5 N is sufficient to obtain reliable test results while also providing greater comfort for patients during sampling.

## Conclusion

The sensitivity of SARS-CoV-2 NAT is positively correlated with higher cell counts in the samples. However, increasing the force during sampling does not lead to improved NAT sensitivity for SARS-CoV-2 positive patients and can result in an opposite effect. A force of 1.5 N is adequate for obtaining reliable test results while ensuring patient comfort. Future research should investigate alternative approaches, such as modification of swab design, to enhance preanalytical sample quality.

## Notes

### Authors’ contribution

The authors Melcher P and Pietsch C contributed equally.

### Author’s ORCID

Peter Melcher: 0000-0002-4747-8488

### Ethical approval 

This study was conducted after approval by the ethics committee of the University of Leipzig (internal registration number: 057/20-ek). 

### Funding 

None. 

### Competing interests 

The authors declare that they have no competing interests.
